# Antioxidant capacity, antimicrobial activities and chemical composition of *Pistacia atlantica *subsp*. kurdica* essential oil

**DOI:** 10.30466/vrf.2018.83910.2103

**Published:** 2019-12-15

**Authors:** Mehdi Fathollahi, Majid Aminzare, Mehran Mohseni, Hassan Hassanzadazar

**Affiliations:** 1 *Department of Food Safety and Hygiene, School of Public Health, Zanjan University of Medical Sciences, Zanjan, Iran;*; 2 *Department of Food and Drug Control, School of Pharmacy, Zanjan University of Medical Sciences, Zanjan, Iran.*

**Keywords:** Antibacterial activity, Disc diffusion, Radical scavenging activity, Minimum inhibitory concentration

## Abstract

This study aimed to evaluate the composition, antioxidant capacity and antibacterial effects of *Pistacia atlantica* subsp. *kurdica* (baneh) essential oil on some important bacteria in food safety. Essential oil was derived using hydro-distillation method of the baneh fruits. Essential oil composition was determined using gas chromatography–mass spectrometry. The 2,2′-azinobis-(3-ethylbenzothiaziline-6-sulfonate; ABTS) and 2,2-diphenyl-1-picrylhydrazyl (DPPH) methods were used to evaluate antioxidant activity and Folin-Ciocalteu method was used to determine total phenolic content of essential oil. The antibacterial effect of the essential oil against six pathogen bacteria was determined using minimum inhibitory concentration, minimum bactericidal concentration and disc diffusion methods. Monoterpene and sesquiterpene hydrocarbons were main compounds of total identified constituents in the essential oil (approximately 93.50% and 5.45%, respectively). The main compounds were α-pinene, myrcene, limonene, β-pinene and γ-terpineol, respectively. The mean concentration of essential oil providing 50.00% inhibition was 7.54 ± 0.01 mg mL^-1^. Dose-dependent and scavenging activities were seen in DPPH and ABTS tests and total phenolic content within test range of concentrations (0.0625 to 4.00 mg mL^-1^). The highest DPPH radical scavenging activity (48.67 ± 0.84%) was seen at a concentration of 4 mg mL^-1^. The responses of essential oil concentrations to ABTS assay were quite similar to the DPPH reaction, especially in higher concentrations. Both antimicrobial methods demonstrated that the essential oil had broader antibacterial effects against the Gram-positive bacteria than the tested Gram-negative bacteria. Essential oil of *Pistachia atlantica *subsp.* kurdica *can be one of the performing essential oils to be used as a preservative in food industry to increase food safety and reduce food pathogens risks.

## Introduction

Oxidative stress can cause proteins, lipids, nucleic acids and cell membranes damages resulting in a number of chronic diseases such as cancer and neurodegenerative and cardiovascular disorders.^1^ Another serious health risk to humans is the consumption of contaminated foods with some pathogens.^2^ Bioactive compounds such as essential oils (EOs) are considered in medicine and pharmaceutical sciences.^3^ The EOs can inhibit the oxidative mechanisms leading to degenerative and food borne diseases due to their phenolic compounds including tannins, phenolic diterpens, flavonoid and phenol acids.^4^^,^^5^ Antibacterial and antioxidant activities of EOs are the reason of their applications as preservative in fresh and processed foods, as natural remedies and pharmaceuticals and in complementary medicine.^6^^-^^8^

Pistacia trees are members of the Anacardiaceae family having more than 11 species and amongst them, *P. vera*,* P. lentiscus *var.* chia *and* P. atlantica* have broad economical and pharmaceutical significances.^9^^,^^10^ It has been recommended that *p. atlantica* has three subspecies in Iran including *kurdica* (baneh), *mutica *(Khanjuk) and *cabulica* (Khandan). The* P. atlantica *subsp*. kurdica* is known as baneh (Ghazvan in Kurdish) distributing in the Mediterranean area especially western Zagros mountain belt of Iran. This distribution is lower in the eastern and central parts of Iran.^8^^,^^9^^,^^11^^,^^12^ Different parts of Pistacia trees and their derivatives such as fruit, leave and resin have been used in an extensive range of traditional medicine as a preservative and breath sweetener as well as to treat gastric ailments and digestive, hepatic and renal diseases.^8^ The fruits of *P. atlantica *subsp*. kurdica* (baneh) are used in different forms by aboriginal people of various parts of Iran including as a flavoring agent in doogh (a cold yogurt beverage mixed with salt), as foods after milling and mixing with other compounds and as a nut after roasting and they are also used to make jam and pickles of unripe fruits. Its hull and kernel oils are used in food frying and gum or oleoresin of baneh trees is used as a main material of chewing gum in Iran known as Kurdish Saghez.^10^

There are several pharmaceutical studies on composition and antioxidant and antimicrobial activities of EO and extracts obtained from different parts of *P. atlantica* species including leaves, fruits, hull and gum.^10^^,^^11^^,^^13^^-^^19^ Due to the scarcity of information about antioxidant and antibacterial activities of the *P. atlantica *subsp*. kurdica* fruits derived EO in the datasets, the aim of this study was to assess *in vitro* antioxidant capacity, antibacterial effects against some important foodborne pathogenic bacteria and chemical composition of this EO to present it as a natural food additive. 

## Materials and Methods

**Collection of plant materials. **Ripen fruits of *P. atlantica *subsp.* kurdica* were collected from different locations of Baneh city (geographic parameters: Altitude 1994 m, latitude 35^◦^57' N and longitude 45^◦^53' E), Kurdistan, Iran at July-August 2016. Plants taxonomical identification was done in the Pharmacognosy and Pharmaceutical Department of Medicinal Plant Research Center, Institute of Medicinal Plants, Karaj, Iran. All fruits were washed and dried at room temperature in the shade for seven days.

**Chemicals**. The 2,2′-azino-bis-(3-ethylbenzothiaziline-6-sulfonate) known as ABTS^+^ and 2,2-diphenyl-1-picrylhydrazyl (DPPH) were obtained from Sigma-Aldrich (St. Louis, MO, USA). Butylhydroxytoluene (BHT), Folin-Ciocalteu reagent and all other chemicals were purchased from Merck Company (Darmstadt, Germany). 

**Essential oil extraction. **The dried fruits of* P. atlantica *subsp.* kurdica* were grinded using mixer grinder and subjected to hydro-distillation by a clevenger apparatus (KOL, Behr, Germany) at 100 ˚C for 3 hr. The distilled EO was isolated and dried with anhydrous sodium sulfate. The dried EO was filtered with 0.22 μm filters and stored in a dark glass sterile bottle at 4.00 ˚C until use.^20^

**Essential oil analysis** *. *The EO composition was determined using gas chromatography–mass spectrometry (GC/MS) method according to Gandomi *et al.*^21^ The EO analysis was implemented using GC/MS method with a gas chromatography apparatus (AGILENT 6890; Agilent Technologies, Santa Clara, USA) equipped with a mass selective detector (AGILENT 5973 series; Agilent Technologies). Analytical conditions were as follows: Capillary column was 30.00 m × 0.25 mm × 0.25 µm and selective mass detector and injector temperatures were 230 and 250 ˚C, respectively. The oven temperature program was 40.00 ˚C at the start which held for 1 min. It raised up to 250 ˚C at a rate of 3.00 ˚C per min. This temperature was held isothermal for 20 min. Carrier gas was helium with 1.00 mL per min flow rate. Electron Ionization (EI) mode (70 eV) in a range of 40.00 - 450 m/z was used for mass spectra recording. The MSD chem Station software (revision E01.01.335; Agilent Technologies) was used for data processing combined with NIST MS Search (ver. 11.0) and the stored Wiley 7 n.1 Mass Computer Library (Wiley-VCH 2001 data software, Weinheim, Germany) by compare acquired retention indices with mass spectra fragmentation. Standard spectra tune was used to obtain better correlation between library and experimental spectra. According to peak area in chromatogram, relative amounts of components were expressed as percentages.

**Antioxidant activity assays. **Two radical scavenging methods were used to evaluate antioxidant activity.

**2, 2'- diphenyl-1-picrylhydrazyl radical scavenging method. **The free radical scavenging ability was determined by the DPPH method described by Aminzare *et al*. with some modifications.^7^ Fifty microliter of provided concentrations (0.0625, 0.125, 0.25, 0.50, 1.00, 2.00 and 4.00 mg mL^-1^) of EO and BHT as a reference antioxidant and control sample were added to tubes containing 2.00 mL of previously prepared methanolic DPPH solution (24.00 μg mL^-1^). The mixtures were shacked and left for 60 min in a dark place at room temperature and the absorbance was measured at 517 nm using methanol as a blank with a spectrophotometer (Novaspec II; Pharmacia LKB, Uppsala, Sweden). The DPPH radical inhibition percentage was calculated using the following formula:

*Inhibition (%) = (A control–A antioxidant/A control) × 100*

The half maximal inhibitory concentration (IC_50_) of EOs concentration was calculated using the graph by plotting percentage of inhibition versus each sample using Pharmacologic Calculation System (Pharm/PCS) software (version 4.0; Springer-Verlag, New York, USA).

**2,2'-azino-bis-3-ethylbenzothiazoline-6-sulphonic acid method. **The discoloration method of ABTS^+^ radicals is based on the reduction of ABTS radical cations following the methods of Thaiponga *et al*. and Arnao *et al*. with some modifications.^22,23^ Stock solutions were 7.40 mM ABTS^+^ and 2.60 mM potassium persulfate solutions. A mixture of two stock solutions in 1/1 quantities was prepared as a working solution which allowed to react and produce ABTS radicals for 16 hr in a dark place at room temperature. Just before use, 1.00 mL of ABTS^+^ solution was diluted with methanol to obtain an absorbance of 0.70 at 734 nm using the spectrophotometer (Pharmacia LKB). One hundred fifty microliter of various concentrations of EO (0.0625, 0.125, 0.25, 0.50, 1.00, 2.00 and 4.00 mg mL^-1^) and BHT as a reference antioxidant and control were added to 2.85 mL of the fresh ABTS^+^ solution and allowed to react for two hr in a dark place at room temperature. Then, the absorbance was measured at 734 nm and the percentage of radical scavenging activity (RSA) was calculated by following formula:

RSA* (%) = (A control–A antioxidant/A control) × 100*

**Total phenolic determination. **The total phenolic content of EO was evaluated using Folin-Ciocalteu methods developed by Hatamnia *et al*. and Raeisi *et al*. with some modifications.^9^^,^^24^ Briefly, 0.10 mL of different concentrations of EO (0.0625, 0.125, 0.25, 0.50, 1.00, 2.00 and 4.00 mg mL^-1^) were added to a mixture of 1.30 mL distilled water plus 100 μL Folin-Ciocalteu reagent and mixed thoroughly. After 6 min, 1.00 mL Na_2_CO_3_ solution (7.00%) was added for neutralization of reaction. After that, it was incubated at room temperature for 90 min and absorbance of the mixtures was recorded at 750 nm using a spectrophotometer (Pharmacia LKB). Alcoholic solution of gallic acid and a mixture of distilled water with reagents were used as standard and blank solutions, respectively. Results were expressed as mg of gallic acid per gram of EO equivalent relative to obtained values of prepared standard curve using determined concentrations of gallic acid. 

**Antibacterial assay. **The minimum inhibitory concentration (MIC), minimum bactericidal concentration (MBC) and disc diffusion methods were used to determine antibacterial activity of EO.

**Microbial strains. **The reference bacterial strains used in this study were *Staphylococcus aureus* (ATCC 25923), *Escherichia coli* (ATCC 15224), *Pseudomonas aeroginosa* (ATCC 15442), *Listeria monocytogenesis* (ATCC 13932), *Shwanella sp.* (PTCC 1711) and *Salmonella enterica* (ATCC 14028) provided from Department of Biotechnology, Iranian Research Organization for Science and Technology (IROST), Tehran, Iran. Intermediate cultures were prepared from the stock cultures according to preparation guideline of IROST to create fresh 18-24 hr cultures before each experiment. Fresh suspensions of tested bacteria were prepared by transfer a loopful from a stock culture to brain-heart infusion (BHI) broth (Merck) and incubation at 35.00 ˚C for 18 to 24 hr. Prepared suspensions were adjusted to an absorbance of 0.10 at 600 nm using a spectro-photometer (Milton Roy Company, Warminster, USA). Bacterial enumeration and colony count were done by duplicate plating from tenfold serial dilution on BHI agar after incubation at 35.00 ˚C for 24 hr. The average number of bacteria in the optical density of 0.10 was obtained equal to 5.00 × 10^8^ CFU mL^-1^.

**Estimation of MIC. **The MIC was determined with broth micro-dilution method recommended by the Clinical and Laboratory Standards Institute (CLSI) with some modifications.^21^ Briefly, two-fold serial dilutions of the stock EO were prepared in BHI broth. Primary concentration of stock EO solution was prepared in 4.00 mg mL^-1^ The concentration of EO dissolved in 10.00% dimethyl sulfoxide (DMSO). To each well of a 96-well micro-plate, 160 μL of BHI broth, 20.00 μL of each inoculum (5.00 × 10^6^ CFU mL^-1^) and 20.00 µL of stock EO solutions were added, respectively. The final concentration of bacteria in each well was 5.00 × 10^5^ CFU mL^-1^. In each strip, a well was selected as a positive control contained 180 μL of BHI broth and 20.00 μL of the inoculum without EO and the last well contained 180 μL of BHI broth and 20.00 μL of EO without any inoculum was selected as a negative control. The micro-plates were incubated at 37.00 ˚C for 24 hr. The MIC is the lowest concentration of the EO preventing visible growth of the bacteria and the MBC is the lowest concentration of EO or an antimicrobial killing a micro-organism and preventing growth on the agar plate. In other word, MBC is the lowest concentration showing a pre-determined reduction (such as 99.90%) compared to MIC dilution. A well containing only the sterile BHI broth medium and a well containing the bacterial culture and DMSO without EO were considered as negative and positive controls, respectively.

**Disc diffusion method. **Determination of microbial susceptibility and confirmation of MICs were performed using the disk diffusion method described in a previous study for pure EO with slight modifications.^25^ Sterile paper discs (6.00 mm diameter) were impregnated with 10.00 μL of each dilution of EO/DMSO (including 0.0625, 0.125, 0.25, 0.50, 1.00, 2.00 and 4.00 mg mL^-1^) and placed on BHI agar plates within one hour after pouring of bacterial cultures. Inoculated plates were then incubated at 37.00 ˚C for 24 hr and the zones of growth inhibition were measured to the nearest mm. In this method, MIC is determined as the lowest concentration causing a visible inhibition zone around the discs compared to control discs (disks impregnated with sterile distilled water). 

**Statistical analysis.** All assays and tests were performed in triplicate. Statistical analysis of data was carried out using SPSS (version 24.0; IBM, Chicago, USA). Data were expressed as mean ± standard deviation of three replicates. Tukey and Duncan tests were used for pair wise comparing (mean ± SD) of the EO with controls.

## Results

**Chemical composition of EO. **The dried Pistacia fruits yielded 0.30-0.40% (v/w) of EO. Thirty compounds were identified in EO as shown in Table 1 representing 98.97% of the total oil. The main compounds were α-Pinene and Myrcene followed by Limonene, β-pinene and γ-Terpineol.

**Table 1:** Chemical composition of baneh fruits essential oil (Gas chromatography–mass spectrometry (GC/MS) analysis)

**Components**	**Amount (%)**	**Retention time (RT)**
**α-Thujene**	0.27	11.15
**α-Pinene**	71.79	11.82
**Camphene**	1.74	12.60
**Thuja-2,4(10)-diene**	0.28	12.82
**β-Pinene**	2.08	14.05
**Myrcene**	9.04	14.64
**α-Phellandrene**	0.23	15.56
**α-Terpinene**	0.16	16.10
**Ortho-Cymene**	0.16	16.60
**Limonene**	2.71	16.77
**β-Phellandrene**	0.32	16.88
**E-β-Ocimene**	0.34	17.64
**γ-Terpinen**	0.17	18.30
**Terpinene**	1.35	19.69
**Para-Cymenene**	0.26	20.14
**Endo-Fenchol**	0.18	21.66
**α-Campholenal**	0.15	22.06
**Trans-Pinocarveol**	0.22	22.75
**γ-Terpineol**	2.02	25.52
**Isobornyl Formate**	0.72	29.53
**E-Caryophyllene**	1.92	35.48
**α-Humulene**	0.23	37.04
**α-Amorphene**	0.20	37.81
**Viridiflorene**	0.20	38.49
**Bicyclogermacrene**	0.07	38.71
**γ-Cadinene**	0.15	39.44
**δ-Cadinene**	0.35	39.58
**3Z-Hexenyl benzoate**	0.57	41.95
**Spathulenol**	0.86	42.15
**2E-Hexenyl benzoate**	0.26	42.58
**Total **	98.97	-

**Free radical-scavenging assay**. DPPH and ABTS free radical scavenging capacities and total phenolic content of *P. atlantica *subsp.* kurdica* fruits EO are shown in Table 2.

The results showed that with increasing concentration from 0.125 to 4.00 mg mL^-1^ of EO, the DPPH and ABTS scavenging abilities were increased significantly (*p* ≤ 0.05) compared to BHT as a reference compound (82.38 ± 0.25%). The IC_50_ or efficient concentration value of the EO (the concentration of compound necessary to decrease 50.00% DPPH activity) was 7.54 ± 0.01 mg mL^-1^ which was calculated graphically (inhibition percent which is plotted versus the logarithm of antioxidant concentration in reaction system). The IC_50_ for the BHT antioxidant was 2.60 ± 0.47 mg mL^-1^. As expected, total phenolic content of EO was increased in higher concentrations of EO. 

**Table 2:** DPPH and ABTS^+^ radicals scavenging activity and total phenolic content of baneh fruits essential oil

**Concentration **	**DPPH scavenging activity (%)**	**ABTS free radical scavenging (%)**	**Total phenolic content (mg gallic acid g** ^-1^ **)**
**0.0625 mg mL** ^-1^	18.10 ± 0.69^a^	17.10 ± 2.20^a^	0.80 ± 0.20^a^
**0.125 mg mL** ^-1^	19.45 ± 0.48^a^	21.56 ± 1.80^b^	1.60 ± 0.40^b^
**0.25 mg mL** ^-1^	26.35 ± 0.51^b^	36.21 ± 0.36^c^	6.70 ± 1.30^c^
**0.50 mg mL** ^-1^	33.50 ± 1.50^c^	52.90 ± 1.43^d^	8.90 ± 3.50^d^
**1.00 mg mL** ^-1^	35.80 ± 1.70^c^	77.30 ± 1.25^e^	16.30 ± 1.80^e^
**2.00 mg mL** ^-1^	42.56 ± 0.89^e^	83.70 ± 0.75^f^	19.10 ± 2.10^f^
**4.00 mg mL** ^-1^	48.67 ± 0.84^f^	85.92 ± 1.30^f^	28.60 ± 3.20^d^
**BHT**	82.38 ± 0.25	99.47 ± 0.30	-

BHT: Butylhydroxytoluene.

Values followed by different letters within the same columns are significantly different (*p *< 0.05) according the Duncan test.

**Antibacterial activity. **The MIC of EO and growth inhibition zone for inhibition of *S. aureus*, *L. mono-cytogenesis*, *Shwanella *sp*.*, *E. coli*, *P.* *aeroginosa* and *S. enterica* were 0.125, 0.50, 0.25, 0.50, 0.25 and 0.50 mg mL^-1^ and 7.33 ± 0.58, 13.33 ± 0.57, 11.67 ± 0.57, 14.33 ± 0.58, 12.33 ± 0.57 and 22.33 ± 0.58 mm, respectively. The MBC of EO required to kill these pathogens and growth inhibition zone at these concentrations were 0.25, 1.00, 0.50, 1.00, 0.50 and 1.00 mg mL^-1^ and 19.20 ± 0.98, 15.67 ± 0.28, 21.33 ± 0.50, 16.67 ± 0.35, 14.33 ± 0.15 and 28.33±0.18 mm, respectively. 

## Discussion

Chemical composition of the fruit's EO of *P. atlantica *subsp*. kurdica* showed that monoterpene and sesquiterpene hydrocarbons (93.50% and 5.45%, respectively) were main compounds of the total identified constituents in the EO (Table 1). Several studies were conducted to evaluate the chemical composition of EOs obtained from different plant parts of the same species or subspecies belonging to the *P. atlantica*, which is shown in Table 3. As illustrated in Table 3, α-Pinene is the main component of all subspecies of *P. atlantica*. Difference in chemical composition of plant's EOs can be attributed to some environmental factors such as season, age, water availability, temperature, altitude and the atmosphere of the harvesting region and other factors such as selected parts of the plant used for EO extraction.^24^

The EO of baneh exhibited dose-dependent and scavenging activities within the test range of concentrations. The highest DPPH radical scavenging activity (48.67 ± 0.84%) was seen at a concentration of 4 mg mL^-1^. Compared to the results of a study implemented by Aliakbarlu *et al*., on some common spices, radical scavenging activity of baneh EO (48.67%) was higher than cinnamon (13.02%), cumin (14.41%), black cumin (33.22%), horsemint (40.10%) and sage (25.11%), but it was lower than *Zataria multifura* (88.63%), clove (90.69%), spearmint (69.45%), coriander (57.72%) and ginger (63.25%) in the same concentration (4.00 mg mL^-1^).^26^ The lowest value of IC_50_ shows the highest antioxidant capacity of the substrate.^4^

**Table 3:** Chemical composition (%) variation in different parts and species of *Pistacia* determined in some researches

**Species of ** ***Pistacia***	**α-Pinene**	**β-Pinene**	**Myrcene**	**Limonene**	**Terpineol**	** Geographical origin**
***atlantica *** **subsp. ** ***kurdica*** ** (Fruits)**	71.79	2.08	9.04	2.71	2.02	Present study, Kurdistan, Iran
***atlantica *** **subsp. ** ***kurdica*** ** (Unknown)**	91.47	2.47	0.48	0.60	-	Kurdistan, Iran ^15^
***atlantica Desf. *** **(Leaves)**	5.54 - 66.61	1.09 - 13.12	0.35	0.62 - 1.62	0.44 - 1.57	Algeria ^4^
***atlantica *** **subsp** **.** *** mutica *** **(Hull)**	20.80	4.20	8.20	8.00	0.30	Fars, Iran ^40^
***atlantica *** **subsp. ** ***kurdica *** **(Gum)**	41.23	6.85	1.18	3.03	0.15	Kurdistan, Iran ^43^

The ABTS^+^ radicals are used to evaluate *in vitro* antioxidant activity of different substrates based on discoloration of intense green of ABTS radicals. Antioxidant activity of both water-soluble and lipid-soluble antioxidants is measurable with this method.^27,28^ According to our literature review, this research is the first study to determine ABTS radical scavenging ability of this EO. The responses of EO concentrations to ABTS assay were quite similar to the DPPH reaction especially in higher concentrations (Table 2). Radicals scavenging activity was higher in ABTS assay than that of DPPH assay. As an important limitation, it may be due to the solubility of DPPH reagent only in organic solvents such as methanol or ethanol.^27^^,^^29^ Aromatic antioxidants of plants can scavenge the ABTS^+^ cations; therefore, inhibition percentage increases with increasing EO concentration.^30^

Hydroxyl groups of phenols have radical's inhibition potential.^31^ These results confirmed that the high phenolic compounds in baneh EO are corresponded to the antioxidant capacity and higher radicals scavenging ability.^32^ Obtained results were matched with the findings of Hatamnia and coworkers.^9^ They have reported the fact that the DPPH scavenging and ferric reducing antioxidant power of extracts are in a phenolic content-dependent manner in all five genotypes of *P. atlantica *subsp.* kurdica* collected from different provinces of Iran.^9^

According to the results of disk diffusion assay, the EO was found to be effective against all tested bacteria at a 10.00 µL of MIC dose. Both methods demonstrated that the EO had broader antibacterial effects against the Gram- positive bacteria (*L. monocytogenes*, *S. aureu*s and *Shwanella *sp*.*) than the tested Gram-negative bacteria (*S. entritidis*,* P. aeroginosa *and* E. coli*). 

Gram-negative bacteria showed higher resistance than Gram-positive bacteria to EOs probably due to their distinguished membrane structure and complexity of their double layer cell membrane. Outer phospholipid membrane of Gram-negative bacteria acts as a barrier and makes the membrane impenetrable to hydrophobic compounds such as EOs.^20^^,^^33^^-^^35^ In Gram-positive bacteria, lack of outer phospholipid membrane facilitates direct contact between lipophilic components and the phospholipid layer of the cell membrane enhancing ion permeability, intracellular constituent excretion and destruction of protein components such as enzymes.^3^^,^^33^^,^^36^^,^^37^

Based on MIC, an acceptable antimicrobial characteristic was seen in the baneh EO in comparison with the other recognized EOs of some medicinal plants used in food systems (Table 4). So far, a few studies were implemented on antibacterial effects of the *P. atlantica *subsp.* kurdica* EO.^15^^,^^38^ Antibacterial evaluations of this EO was conducted for the first time on the tested bacteria strains. Sharifi and Hazel have shown antibacterial activities of *P. atlantica *subsp.* kurdica* EO extruded of trunk exudate (gum) on nine strains of *Helicobacter pylori* and some Gram-negative and Gram-positive bacteria with MIC ranging from 500 to 1000 mg mL^-1^.^39^ Rezaei *et al*. have shown significant antibacterial activities of *P. atlantica *subsp*. mutica* against *S. aureus* and *E. coli* with MIC value ranges of 6.00 - 12.50 μg mL^-1^, respectively.^40^ Relationship between the anti-microbial effects of some EOs and presence of phenolic compounds such as monoterpens has been proven previously.^41^ Due to presence of phenolic compounds, sensitivity of the tested bacteria to this EO can be expected. However, attributing of antimicrobial effect of an EO to one or specific components is not appropriate.^42^

**Table 4:** Minimal inhibitory concentration (mg mL^-1^) of some recognized essential oils (EOs) against the four tested bacteria

**Essential oil**	***E. coli***	***S. entritidis***	***S. aureus***	***L. monocytogenes***	**References**
**Baneh EO**	0.50	0.50	0.125	0.50	Present study
**Clove**	1.25	0.625	0.625	0.625	
**Rosemary**	1.25	2.50	0.3125	0.3125	
**Cinnamon**	2.50	2.50	0.625	0.625	
**Clove**	1.25	1.25	-	0.625	
**Ginger**	> 40	> 40	-	5.00	
**Thyme**	0.25	11.00	-	0.50	
**Thyme** ^†^	2.00, 8.00, 16.00*^†^	-	0.50, 2.00, 4.00^†^	-	
**Origanum**	1.56	3.125	1.56	1.56	

* *E. coli* O157 H7; ^† ^Essential oils of *Zataria multiflora Boiss*. from different parts.

Since natural compounds are considered increasingly in food industry, pharmaceuticals and cosmetics, finding alternatives for synthetic preservatives in food is one of the important interests in food science. Recently conducted studies have shown that EOs have particular functional properties as antioxidants and antimicrobial compounds.^1^^,^^3^ Considering chemical composition and antioxidant and antimicrobial activities of the *P. atlantica* subsp. kurdica fruit’s EO and its moderate capacity to scavenge free radicals, it can be one of the performing EOs to be used as a preservative in food industry to increase food safety and reduce food pathogens risks. However, more studies are needed to evaluate its mode of action as well as pharmacological and organoleptic effects in food systems.

## References

[1] Rezaie M, Farhoosh R, Pham N (2016). Dereplication of antioxidant compounds in Bene (Pistacia atlantica subsp mutica) hull using a multiplex approach of HPLC–DAD LC–MS and (1)H NMR techniques. J Pharm Biomed Anal.

[2] Hussain AI, Anwar F, Hussain Sherazi ST (2008). Chemical composition, antioxidant and antimicrobial activities of basil (Ocimum basilicum) essential oils depends on seasonal variations. Food Chem.

[3] Raeisi M, Tajik H, Yarahmadi A (2015). Antimicrobial effect of cinnamon essential oil against Escherichia coli and Staphylococcus aureus. Health Scope.

[4] Gourine N, Yousfi M, Bombarda I (2010). Antioxidant activities and chemical composition of essential oil of Pistacia atlantica from Algeria. Ind Crop Prod.

[5] Aminzare M, Amiri E, Abbasi Z (2017). Evaluation of in vitro antioxidant characteristics of corn starch bioactive films impregnated with Bunium persicum and Zataria multiflora essential oils. Annu Res Rev Biol.

[6] Aminzare M, Hashemi M, Hassanzadazar H (2017). Anti-bacterial activity of corn starch films incorporated with Zataria multiflora and Bunium persicum essential oils. Annu Res Rev Biol.

[7] Aminzare M, Aliakbarlu J, Tajik H (2015). The effect of Cinnamomum zeylanicum essential oil on chemical characteristics of Lyoner-type sausage during refrigerated storage. Vet Res Forum.

[8] Bozorgi M, Memariani Z, Mobli M (2013). Five Pistacia species : A review of their traditional uses, phytochemistry, and pharmacology. Sci World J.

[9] Hatamnia AA, Abbaspour N, Darvishzadeh R (2014). Antioxidant activity and phenolic profile of different parts of Bene (Pistacia atlantica subsp. kurdica) fruits. Food Chem.

[10] Taran M, Sharifi M, Azizi Eet al (2010). Antimicrobial activity of the leaves of Pistacia khinjuk. J Med Plant.

[11] Hatamnia AA, Rostamzad A, Hosseini M (2015). Antioxidant capacity and phenolic composition of leaves from 10 Bene (Pistacia atlantica subsp kurdica) genotypes. Nat Prod Res.

[12] Farhoosh R, Tavakoli J, Hadad-Khodaparast MH (2008). Chemical composition and oxidative stability of kernel oils from two current subspecies of Pistacia atlantica in Iran. ‎ J Am Oil Chem Soc.

[13] Farhoosh R, Tavassoli-Kafrani MH, Sharif A (2011). Anti-oxidant activity of the fractions separated from the unsaponifiable matter of bene hull oil. Food Chem.

[14] Ghalem BR, Mohamed B (2009). Essential oil from gum of Pistacia atlantica Desf: Screening of antimicrobial activity. Afr J Pharm Pharmacol.

[15] Hesami G, Bahramian S, Fatemi A (2014). Effect of Pistacia atlantica subsp kurdica essential oil and acetic acid on botrytis cinerea growth in culture media and strawberry fruits. Bull Env Pharmacol Life Sci.

[16] Hosseini F, Adlgostar A, Sharifnia F (2013). Antibacterial Activity of Pistacia atlantica extracts on Streptococcus mutans biofilm. Int Res J Biological Sci.

[17] Peksel A (2008). Antioxidative properties of decoction of Pistacia atlantica Desf. leaves. Asian J Chem.

[18] Salimi F, Shafaghat A, Sahebalzamani H (2011). α-Pinene from Pistacia atlantica Desf Subsp Kurdica (Zohary) Rech. F. Der Chemica Sinica.

[19] Tohidi M, Khayami M, Nejati V (2011). Evaluation of antibacterial activity and wound healing of Pistacia atlantica and Pistacia khinjuk. J Med Plants Res.

[20] Raeisi M, Tajik H, Aminzare M (2016). The role of nisin monolaurin and EDTA in antibacterial effect of Rosmarinus Officinalis L and Cinnamomum Zeylanicum blume essential oils on foodborne pathogens. J Essent Oil Bear Pl.

[21] Gandomi H, Abbaszadeh S, Jebellijavan A (2014). Chemical constituents, antimicrobial and antioxidative effects of Trachyspermum ammi essential oil. J Food Process Preserv.

[22] Thaiponga K, Boonprakoba U, Crosbyb K (2006). Comparison of ABTS, DPPH, FRAP, and ORAC assays for estimating antioxidant activity from guava fruit extracts. J Food Compos Anal.

[23] Arnao MB, Cano A, Acosta M (2001). The hydrophilic and lipophilic contribution to total antioxidant activity. Food Chem.

[24] Raeisi M, Hashemi M, Aminzare M (2016). Comparative evaluation of phytochemical, antioxidant, and antibacterial properties from the essential oils of four commonly consuming plants in Iran. J Food Qual Hazards Control.

[25] Iturriaga L, Olabarrieta I, de Maranon IM (2012). Antimicrobial assays of natural extracts and their inhibitory effect against Listeria innocua and fish spoilage bacteria, after incorporation into biopolymer edible films. Int J Food Microbiol.

[26] Aliakbarlu J, Sadaghiani SK, Mohammadi S (2013). Comparative evaluation of antioxidant and anti-food-borne bacterial activities of essential oils from some spices commonly consumed in Iran. Food Sci Biotechnol.

[27] Ehsani A, Hashemi M, Afshari A (2016). Antioxidant activity of Echinophora platyloba DC essential oil: A comparative study on four different methods. Iran J Vet Sci Technol.

[28] Erkan N, Ayranci G, Ayranci E (2008). Antioxidant activities of rosemary (Rosmarinus Officinalis L) extract, blackseed (Nigella sativa L) essential oil, carnosic acid, rosmarinic acid and sesamol. Food Chem.

[29] Zeng LB, Zhang ZR, Luo ZH (2011). Antioxidant activity and chemical constituents of essential oil and extracts of Rhizoma Homalomenae. Food Chem.

[30] Proestos Ch, Lytoudi K, Mavromelanidou OK (2013). Antioxidant capacity of selected plant extracts and their essential oils. Antioxidants (Basel).

[31] Zangiabadi M, Sahari MA, Barzegar M (2012). Zataria multiflora and Bunium persicum essential oils as two natural antioxidants. J Med Plants.

[32] Cai Y, Luob Q, Sunc M (2004). Antioxidant activity and phenolic compounds of 112 traditional Chinese medicinal plants associated with anticancer. Life Sci.

[33] Delamare APL, Moschen-Pistorello IT, Artico L (2007). Antibacterial activity of the essential oils of Salvia officinalis L and Salvia triloba L cultivated in South Brazil. Food Chem.

[34] Marongiu B, Piras A, Porcedda S (2007). Supercritical CO2 extract of Cinnamomum zeylanicum: Chemical characterization and antityrosinase activity. J Agric Food Chem.

[35] Teixeira B, Marques A, Ramos C (2013). Chemical composition and antibacterial and antioxidant properties of commercial essential oils. Ind Crops Prod.

[36] Nostro A, Germano MP, D’ Angelo V (2000). Extraction methods and bioautography for evaluation of medicinal plant antimicrobial activity. Lett Appl Microbiol.

[37] Tajik H, Aminzare M, Mounesi Raad T (2015). Effect of Zataria multiflora Boiss essential oil and grape seed extract on the shelf life of raw buffalo patty and fate of inoculated Listeria monocytogenes. J Food Process Preserv.

[38] Khodavaisy S, Rezaie S, Noorbakhsh F (2016). Effects of Pistacia atlantica subsp kurdica on growth and aflatoxin production by Aspergillus parasiticus. Jundishapur J Microbiol.

[39] Sharifi MSH, Hazel SL (2011). GC-MS analysis and antimicrobial activity of the essential oil of the trunk exudates from Pistacia atlantica kurdica. J Pharm Sci Res.

[40] Rezaie M, Farhoosh R, Sharif A (2015). Chemical composition, antioxidant and antibacterial properties of Bene (Pistacia atlantica subsp mutica) hull essential oil. J Food Sci Technol.

[41] Saei-Dehkordi SS, Tajik H, Moradi M (2010). Chemical composition of essential oils in Zataria multifloraBoiss From different parts of Iran and their radical scavenging and antimicrobial activity. Food Chem Toxicol.

[42] Cavar S, Maksimovic M, Vidic D (2012). Chemical composition and antioxidant and antimicrobial activity of essential oil of Artemisia annua L from Bosnia. Ind Crops Prod.

[43] Minaiyan M, Karimi F, Ghannadi A (2015). Anti-inflammatory effect of Pistacia atlantica subsp kurdica volatile oil and gum on acetic acid-induced acute colitis in rat. Res J Pharmacogn.

[44] Ibrahim FA, Bellail AA, Hamad AM (2017). Antimicrobial activities and chemical composition of the essential oil of Origanum majorana L growing in Libya. Int J Pharm Pharm Sci.

